# P53 and PIK3CA Mutations in KRAS/HER2 Negative Ovarian Intestinal-Type Mucinous Carcinoma Associated with Mature Teratoma

**DOI:** 10.1155/2020/8863610

**Published:** 2020-07-22

**Authors:** Sarah Bouri, Philippe Simon, Nicky D'Haene, Xavier Catteau, Jean-Christophe Noël

**Affiliations:** ^1^Department of Pathology, Erasme University Hospital, Université Libre de Bruxelles, Brussels, Belgium; ^2^Centre Universitaire Inter Regional d'Expertise en Anatomie Pathologique Hospitalière (CurePath), Jumet, Belgium; ^3^Department of Gynecology, Erasme University Hospital, Université Libre de Bruxelles, Brussels, Belgium

## Abstract

Primary ovarian intestinal-type mucinous carcinomas associated with mature teratoma are rare and represent less than 3% of all primary ovarian neoplasms. The molecular profile of these tumors is still controversial. We report here the first case of mucinous ovarian tumor in which mutation of the PIK3CA and P53 genes could be demonstrated by the next generation sequencing technique without KRAS mutation or HER2 amplification. Our data suggest that these mucinous carcinoma variants probably present an extremely complex molecular biology profile that should be known in the future to stratify therapeutic outcomes and potential targeted therapies, particularly in recurrent disease.

## 1. Introduction

The last years, a dualistic approach to ovarian carcinomas (type I and type II tumors) has emerged based on both morphological criteria and the molecular biology profile of these tumors including inactivations or mutations of various genes. In particular, the P53 gene is characteristically mutated in type II tumors including high-grade serous carcinomas [1]. By opposition, mucinous carcinomas of the ovary, which account for 3% of all primary ovarian neoplasms, are classically classified in the type I group [1, 2]. Their origin is uncertain but they can originate from either ovarian surface epithelium, metaplastic ovarian cysts, Brenner tumors, or teratomas [3–5]. Classically, two histological variants of mucinous borderline tumors have been described including either intestinal type (the most frequent) or endocervical type [6, 7].

Genotypically, KRAS gene mutation is the most common gene alteration found in these tumors. KRAS mutations have been described as well in benign than in malignant mucinous ovarian tumors, suggesting that it probably plays a major role in the progression from benign to malignant phenotype [2, 8–10]. Other mutations including PIK3CA, PTEN, BRAF, EGFR, KIT, STK11, CDKN2A, and P53 genes have also been described mostly in invasive mucinous carcinomas suggesting that these mutations take place belatedly in ovarian mucinous carcinogenesis [2, 8]. In addition, HER2 amplification is also common in mucinous carcinomas, occurring in about 20% of cases and suggested HER2-targeted therapy as a potential option for HER2 amplified advanced or recurrent disease [11]. Mucinous carcinomas without KRAS mutation and/or HER2 amplification (KRAS/HER2: -) occur with a variable frequency according to the populations tested with an average of 10% in agreement with two previous studies [8, 10]. Among the HER2/KRAS negative cancers, only one study was able to demonstrate on four tested cases, the presence of various additional mutations (CDKN2A, P53, BRAF, FGFR2, and STK11) [8]. However, to the best of our knowledge, we report here the first case of ovarian mucinous carcinoma associated with mature teratoma showing both PIK3CA and P53 genes mutation without KRAS mutation or HER2 amplification. These data are discussed according to the highlights of the literature.

## 2. Case Presentation

**Figure 1:**
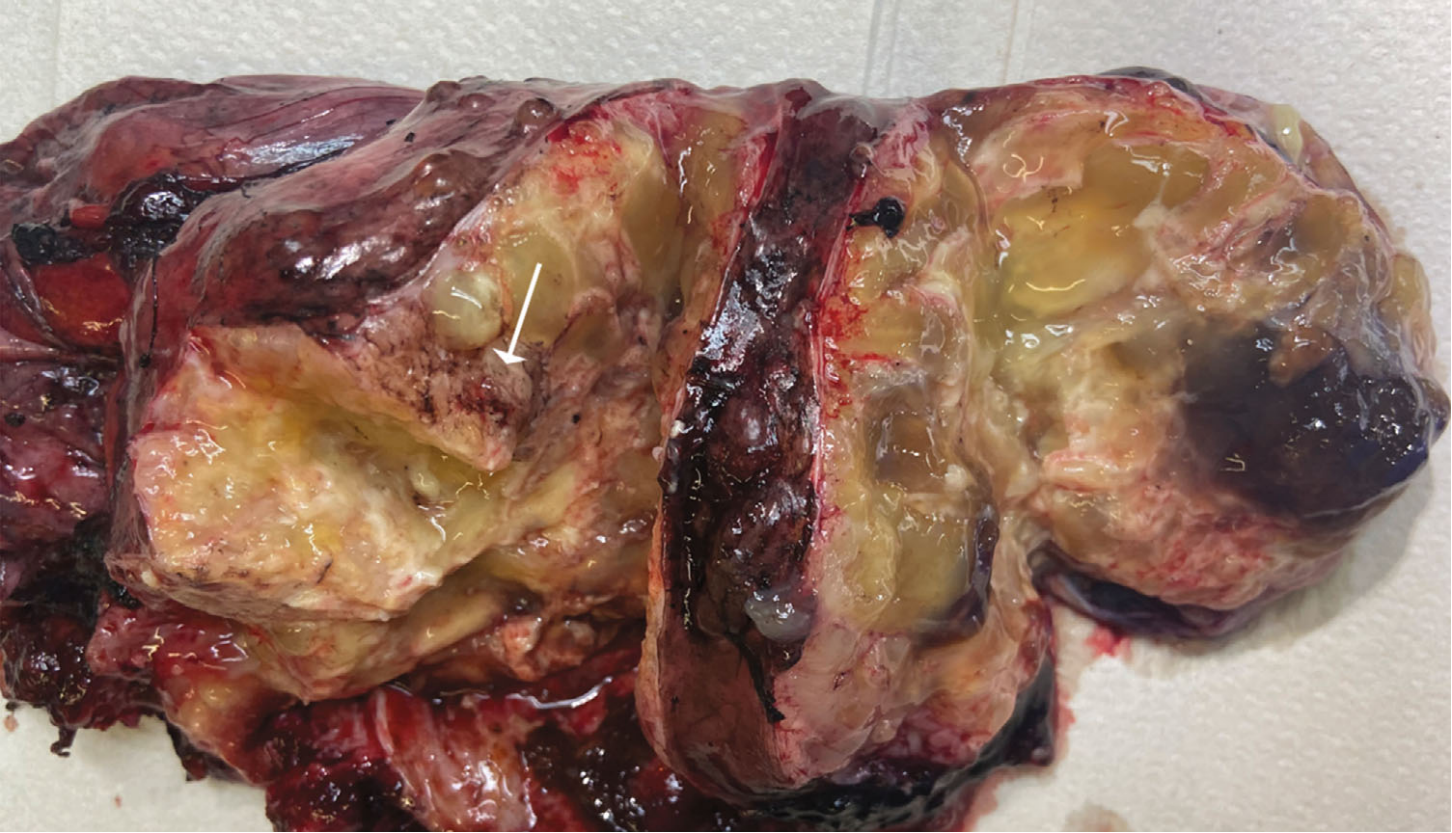
Macroscopic aspects of the tumor. Cystic and solid mucinous tumor with viscous content. Note the presence of solid area with osseous tissue (arrow). The capsule is intact.

A 47-years-old female with a past history of sarcoidosis and endometriosis was referred to the gynecological consultation of Erasme University Hospital for an acute left lower abdominal painful mass at the gynecologic examination. Abdominal computed tomography (CT) examination showed a 17 cm left adnexal cystic lesion containing thin septa highly suspicious of malignancy. This tumor was surgically resected and frozen section examination suggested the diagnosis of a (mucinous tumor possibly invasive). Therefore, due to the age of the patient (without desire of fertility), a total hysterectomy with contralateral salpingo-oophorectomy and omentectomy was performed. Macroscopic examination revealed a complex multilocular 17 cm left ovarian tumor, with yellowish mucinous component and solid areas with some of them containing osseous tissue. There was no disruption on the external surface of the tumor (Figure 1).

**Figure 2:**
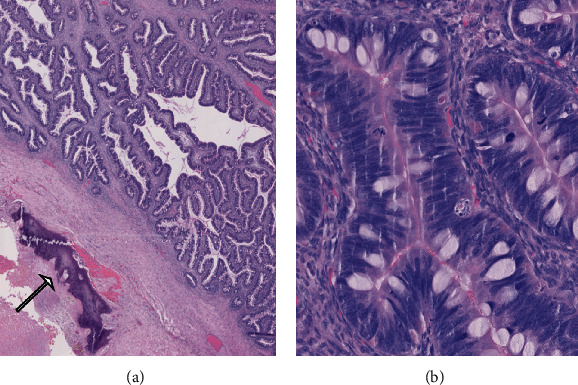
Pathologic aspects of the ovarian intestinal-type mucinous carcinoma at low power view. Note the presence of residual bone tissue (arrow) (a). At a high power view, the glands are lined by columnar and goblet cells with moderate atypia and brisk mitotic activity (b).

**Figure 3:**
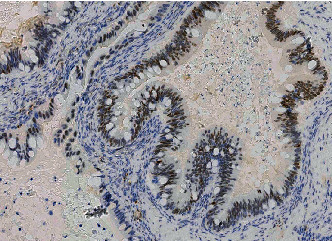
Positive immunohistochemistry of the tumoral cells for SATB2.

Microscopically, the tumor was heterogeneous with both benign, borderline, and invasive mucinous components (expansile and destructive patterns of invasion). Cytologically, the glands are lined by columnar cells with numerous goblet cells. Moderate to severe atypia and brisk mitotic activity were noted (Figures 2). By immunohistochemistry, as we have previously described, the tumoral cells were positive for CK7, CK20, CDX2, PAX8, P53 (diffuse (diffuse/mutated staining), SATB2 and negative for WT1, ER, PR, p16 [12] (Figure 3). HER 2 staining was moderately positive (++) but FISH examination was negative. Immediately adjacent to the malignant glandular component, residual teratomatous bone tissue was observed (Figures 2). Endometriotric lesions were also observed at the periphery of the tumor but no Whaltard cell nest.

No implant was noted in the epiplon and no tumoral cell was present in the peritoneal washing. The contralateral ovary, the rest of the gynecological examination, and the appendix were unremarkable. The tumor was staged pT1a according to the UICC 2017. No complementary treatment was applied and to date with a follow-up of 3 months the patient was disease free.

After the dissection of the tumoral component, gene mutation testing has been performed by next generation sequencing (NGS), as we have previously validated, with a panel of 16 genes described in Table 1 [13, 14]. Next generation sequencing (NGS) is a DNA sequencing technology technique that enables massive gene sequencing in parallel.

Two mutations were found: R248Q (exon 7) mutation of the P53 gene and E545K (exon 9) mutation of the PIK3CA gene.

## 3. Discussion

True primary mucinous ovarian carcinomas are rare tumors and account for less than 5% of all ovarian carcinomas. The molecular profile of these tumors remains still controversial but recent data suggest that the most frequent mutations encountered are KRAS, CDKN2A, and P53 gene mutations [2, 8, 9]. Generally, the mucinous carcinoma of intestinal-type is associated with benign and mucinous areas and suggests a continuum between these components where KRAS gene mutations are early events. By contrast, P53 gene mutations and/or HER2 amplification seem to be acquired later in malignant transformation. Mucinous carcinomas without KRAS mutation and/or HER2 amplification (KRAS/HER2: -) account for about 10% of all mucinous carcinomas. In the 4 cases clearly documented, the mutations observed in these molecular variants were, respectively, CDKN2A (two cases), P53 (one case), BRAF (one case), FGFR2 (one case), and STK11 (one case) [8]. We describe here the first case with both associated P53 and PIK3CA mutations. Our results lead to several points for consideration. Firstly, classically, ovarian carcinomas have been classified in agreement with a dualistic model according to their histology, clinical evolution but also to their molecular changes [6, 15]. The type 1 tumors included low-grade serous carcinomas, malignant Brenner tumors, clear cell carcinomas, seromucinous carcinomas, endometrioïd carcinomas, and mucinous carcinomas, and the type 2 included high-grade serous carcinomas, undifferentiated carcinomas, and malignant mixed Mullerian tumors [6, 15]. From a molecular point of view, P53 mutations have been demonstrated to be a key event in the pathogenesis of type 2 ovarian tumors and the latest are clearly more aggressive [15]. However, recent molecular analyses suggest that like in the present case, P53 mutations are not so rare in mucinous carcinomas and occur in at least 55% of cases. They are not to date associated with poorer clinical outcomes [2, 8, 9].

Secondly, mucinous ovarian carcinomas of intestinal type associated with teratoma remain uncommon because like in the present case, teratomatous components represent frequently a small part of the tumors and require careful and precise examination in order to be detected [5]. Classically, these tumors occur in younger patients (less than 45 years) and are typically SATB2 positive by immunohistochemistry, by opposition to Brenner tumor-associated carcinomas, which are negative for SATB2 [15]. Due to their rarity, the molecular profiles of intestinal mucinous carcinomas associated with teratoma remain controversial. P53 mutations have been demonstrated in about 20% of cases but PIK3CA mutations have not been yet described [15]. Some authors have also postulated that in addition to the SATB2 status, RNF43 mutations could be a frequent event in teratomatous associated carcinoma. Unfortunately, this gene was not available in our NGS panel [15].

Lastly, if the prognosis of stage 1 mucinous carcinomas remains excellent especially in tumors with an expansile pattern, infiltrative subtypes are more aggressive with local recurrences and/or lymph node involvements and classical adjuvant therapies (mainly carboplatin, paclitaxel,…) give unconstant effects [10]. Therefore, personalised molecular therapeutic strategies (antiestrogens, HER2 targeted therapies, RAS/RAF inhibitors, KRAS inhibitors, P53 reactivators, PI3-kinase inhibitors,…) will become crucial in the future as an alternative/complement to conventional chemotherapy [2, 10]. These last years, in ovarian carcinomas associated with BRCA1/2 mutations, poly (ADP-ribose) polymerase inhibitors (PARP inhibitors) (olaparib, niraparib, veliparib,…) demonstrated evident improvements in progression-free survival [16–18]. Nevertheless, BRCA 1 or 2 mutations or homologous recombinant deficiency are not associated with mucinous carcinomas [2]. Early data in vitro concerning the anti-EGFR monoclonal antibody cetuximab carry out antiproliferative activity only in mEOC cell lines, which did not harbor KRAS mutations and could also constitute promising options [2, 18]. Otherwise, MEK and PI3K inhibitors in patients with KRAS mutations, encountered in more than 40% of ovarian mucinous carcinomas, have been proposed as potential targeted therapies [2, 19]. Lastly, it has been proposed that the PRIMA-1 analog APR 246, a small molecule, which is able to restore both the wild-type conformation and function to mutant P53, could provide attractive new therapies in mutated P53 tumors such as here [2, 18].

These data support that full characterization of mucinous carcinomas including clinical, pathological but also molecular biologic aspects will be indispensable in the near term to stratify therapeutics options.

## Figures and Tables

**Table 1:** Cancer hotspot panel used by NGS.

AKT1	DICER1	FOXL2	POLE
BRAF	ERBB2	KRAS	PTEN
CDKN2A	FBXW7	PIK3CA	RB1
CTNNB1	FGFR2	PIK3R1	TP53
